# Synergistic killing effects of homoharringtonine and arsenic trioxide on acute myeloid leukemia stem cells and the underlying mechanisms

**DOI:** 10.1186/s13046-019-1295-8

**Published:** 2019-07-15

**Authors:** Ming Tan, Qian Zhang, Xiaohong Yuan, Yuanzhong Chen, Yong Wu

**Affiliations:** 1 Fujian Institute of Hematology, Fujian Provincial Key Laboratory on Hematology, Fujian Medical University Union Hospital, 29 Xinquan Road, Fuzhou, 350001 Fujian China; 2 Fujian Medical University graduate school, 1 Xuefu North Road, Fuzhou, 350112 Fujian China

**Keywords:** Homoharringtonine, Arsenic trioxide, LSC, Notch, Xenograft leukemia model

## Abstract

**Background:**

At present, it is generally believed that leukemia stem cells are the source of AML, so the killing of leukemia stem cells has become important. Previous studies have suggested that HHT combined with ATO can synergistically kill U937 cells, and HHT has also demonstrated the ability to kill leukemia stem cells. We evaluated whether HHT combined with ATO can systematically kill leukemia stem cells (LSCs) and explored the synergistic effect and molecular mechanism.

**Methods:**

CCK-8 was used to detect cell viability. The changes of cell cycle (PI staining), apoptosis (Annexin V/PI) and surface markers (CD34, CD38, CD96, CD45) were detected by flow cytometry. The cells of CD34+ primary leukemia and CD38- KG-1, and TF-1 were separated by flow cytometry. High-throughput mRNA sequencing was used to analysis mRNA level changes after the application of the two drugs. Western blot was used to verify the changes of pathway protein expression. NRG mice were used as the receptor of xenograft model. Histological H&E staining assess the invaded ability of leukemia cells, and laser scanning confocal microscopy evaluated the molecule markers change.

**Results:**

HHT and ATO synergistically killed KG-1 (CD34^+^/CD96^+^/CD38^+^/^−^) and Kasumi-1 (CD34^+^/CD38^−^) cells. Their combination had a stronger effect of inducing apoptosis and blocking the cell cycle than HHT or ATO administrator alone, meanwhile significantly reducing the numbers of LSCs. Further, CD34^+^CD38^−^ cells in KG-1, KG-1a, TF-1, and primary leukemia cells were more sensitive to HHT and ATO. High-throughput mRNA sequencing suggested that HHT alone could significantly upregulate molecules related to the Notch, P53, and NF-κB signaling pathways. When combined with ATO, HHT further upregulated P53, whereas HHT-induced NF-κB pathway activation was significantly suppressed. Western blot analysis verified the change of protein expression in the above pathways and further demonstrated that GSI, could eliminate these effects. In vivo, HHT combined with ATO significantly reduced the LSC burden, and weakened the expression of LSC markers.

**Conclusions:**

This is the first evidence that HHT combined with arsenic can synergistically kill LSCs in vitro and in vivo, along with identification of the underlying mechanism, highlighting a potentially effective treatment strategy.

**Electronic supplementary material:**

The online version of this article (10.1186/s13046-019-1295-8) contains supplementary material, which is available to authorized users.

## Background

Acute myeloid leukemia (AML) remains a clinical treatment challenge. Although multi-drug combinational chemotherapy can reduce the remission risk, relapse nevertheless is common. Accumulating evidence points to leukemia stem cells (LSCs) as the root of relapse [1, 2]. Thus, inducing damage to LSCs would be an effective approach to reduce the relapse rate of AML.

LSCs are a specific subgroup of AML cells. The current paradigm states that LSCs of AML are part of the CD34-positive and CD38-negative cell population and thus can be distinguished by various molecular markers [3]. CD96 has recently been applied for the identification of LSCs among CD34^+^/CD38^−^ AML cells [4]; however, there are few studies on this marker. Moreover, the multitudinous cell lines KG-1, KG-1a, Kasumi-1, and TF-1 also contain CD34^+^/CD38^−^ AML cells, with essentially all of these lines being CD34^+^. Accordingly, these cells are most widely used to study LSCs [5, 6].

Homoharringtonine (HHT) and arsenic trioxide (ATO) have been traditionally used in the treatment of AML in China separately; however, little is known about the effects of their combination. The first report of their combined use was conducted in the multiple myeloma cell line RPMI8226 [7]. Subsequently, these two agents were shown to exhibit a synergistic effect in killing AML cells [8]. However, the synergistic anti-proliferative effects of HHT and ATO on LSCs, and the underlying mechanism, remain to be clarified. Thus, we examined the effects of these two agents on LSCs using the cell lines listed above as well as in a xenograft model in vivo. We further explored the potential effects of these agents alone and in combination on the Notch, p53, and nuclear factor-κB (NF-κB) pathways as a candidate mechanism.

Notch is a cell-surface receptor forming a heterodimer comprising extracellular and transmembrane subunits and is typically expressed in both normal hematopoietic cells and leukemia cells [9]. The main effect of Notch is the induction of stem cell proliferation, although recent studies have identified various roles according to cell and disease types. In lymphopoiesis, Notch1 and Notch2 affect T and B cell differentiation and proliferation, respectively; however, their roles in myeloid lineages, especially in LSCs, are not fully clarified [9]. P53 and p21 are important tumor suppressor proteins, and p53 induces the expression of p21 [10]. HHT has been shown to enhance p53/p21 expression in *FLT3-ITD* mutant AML [11], but this effect in LSCs and the consequence for cell proliferation are not well understood. Moreover, p53/p21 expression is induced by Notch ligand in myeloid lineage cells overexpressing Notch/Hes1 [12]. Thus, this effect of HHT on Notch upregulation and subsequent p53/p21 pathway activation might also underlie the killing mechanism of LSCs.

NF-κB is a transcription factor that is constitutively activated in primitive AML cells, and its expression can be reduced by HHT in multiple myeloma cells, while ATO was shown to suppress NF-κB activation in mantle cell lymphoma cells [13–15]. Like p53, the NF-κB pathway is also a target of Notch downstream signaling [9]. However, the effects of HHT and ATO on the NF-κB pathway in LSCs are unknown.

Here, we used CD34^+^/CD38^−^ KG-1 and Kasumi-1 cells along with CD34^+^ primary-cultured cells from patients with AML to investigate the synergistic effect of HHT and ATO in LSCs in vitro*.* In addition, NRG mice injected with KG-1 cells were used as an in vivo xenograft model to investigate the effects of treatment with HHT and ATO alone or in combination. Overall, we demonstrate a synergistic effect of HHT and ATO, inducing greater damage to LSCs in vitro and in vivo than these two drugs using alone. and highlight a link between activation of the tumor suppressor P53 pathway and inhibition of the NIK and NF-κB pathways. These findings provide insight into the pathogenesis of AML, while highlighting key molecules to effectively target LSCs and reduce the risk of remission.

## Methods

### Primary patient and cell lines culture

Mononuclear cells were extracted with a Nicoll–plaque (Haoyang, Tianjin, China) gradient centrifugation method from bone marrow blood samples of patients newly diagnosed with AML (*n* = 7) and samples of benign anemia patients and of allotransplantation patient whose bone marrow showed complete remission (three samples in total), which were preserved when drawing blood for diagnosis after obtaining informed consent. Patients data was listed in Additional file 9: Table S1. Primary cells were cultured in serum-free medium (stemspan™, STEMCELL, Vancouver, Canada) with CC110-cytokine cocktail (Flt3L, IL-6, IL-3, SCF, stemspan™, STEMCELL, Vancouver, Canada). Kasumi-1, TF-1, THP-1 and HEL cells (purchased from ATCC) were cultured in RPMI-1640 medium (Hyclone, Thermo Scientific, Waltham, MA, USA) with 10% fetal bovine serum (Gibco, Life Technologies, Grand Island, NY, USA); 2 ng/ml granulocyte macrophage-colony stimulating factor was added to the medium of TF-1 cells. KG-1 cells and KG-1a were purchased from Kunming Institute of Zoology, Chinese Academy of Science and ATCC respectively, and cultured in IMDM (HyClone) with 20% fetal bovine serum (Gibco). All cells were maintained in a 37 °C incubator with 5% CO_2_.

### Cell sorting

Primary CD34^+^ cells and KG-1, TF-1, CD38^−^, or CD38^+^ cells were sorted by staining with CD34-allophycocyanin (CD34–APC; BD Biosciences, Pharmingen, San Diego, CA, USA) and CD38-APC (BD Biosciences), respectively, using flow cytometry on the FACS Aria II system.

### Cell viability

Cells (5 × 10^4^ cells/ml for KG-1 and Kasumi-1 cells, and 5 × 10^5^ cells/ml for primary leukemia cells; these same concentrations were used for subsequent Kasumi-1 and primary leukemia cells experiments) were treated with indicated HHT (Minsheng pharma, Hangzhou, China), ATO (As_2_O_3_) (Harbin Yida Pharmaceutical Co. Ltd., Harbin, China), or their combination (dissolved in complete culture medium) at 37 °C in a 96-well plate to a final volume of 100 μl culture medium. For the combined treatment, 1 × 10^5^ KG-1 cells/ml were used (the same concentrations were used for subsequent experiments). Subsequently, 10 μl of CCK-8 reagent (Signalway antibody; College Park, MD, USA) was added to each well, and incubated at 37 °C for 4 h. The absorbance was scanned with a BioTek SYNERGY/HTX multi-mode reader at 450 nm, and the half-maximal inhibitory concentration (IC_50_) of the drugs was calculated to determine the cell viability and cell growth inhibition rate as previously described [16].

The combination index (CI) was calculated to determine the synergistic cytotoxicity according to the classic isobologram equation: CI = (D)1/(Dx)1 + (D)2/(Dx)2, where Dx is the concentration of one drug that produces the effect, and (D)1 and (D)2 are the concentrations of the two drugs in combination that produce the same effect. CI = 1 indicates an additive effect, CI < 1 indicates synergy, and CI > 1 reflects antagonism between two drugs.

### Hoechest staining

Forty-eight hours after treatment with HHT, ATO, or their combination, the cells were harvested, washed three times with phosphate-buffered saline (PBS), and prepared as a smear. The cells were immobilized with 4% paraformaldehyde (Biosharp, Hefei, Anhui, China) for 30 min, air-dried, washed with PBS, and permeabilized with 0.5% Triton X-100 (Solarbio, Beijing China) for 30 min. After washing with PBS again, the cells were stained with Hoechst 33342 (Cell Signal Technology, Danvers, MA, USA) for 30 min at room temperature to detect apoptosis; the nuclear morphology change was immediately evaluated using a fluorescence microscope (Olympus, Tokyo, Japan) after a final wash with PBS.

### Flow cytometry

Cell cycle, cell apoptosis, and cell surface antigens were detected using flow cytometry. In brief, 48 h after treatment of KG-1, Kasumi-1, KG-1a, TF-1, or primary cells, the cell density was adjusted according to the cell viability assay and prepared for cell cycle analysis as described in our previous report [17]. Cells were stained with Annexin V-fluorescein isothiocyanate (FITC)/propidium iodide (PI) (BD Pharmingen, San Diego, CA, USA) to detect the apoptosis state in accordance with the manufacturer specifications. The CD34^+^/CD38^−^ population (Kasumi-1 cells) or CD34^+^/CD38^−^/CD96^+^ population (KG-1 cells) was obtained by incubating with CD34–FITC (Biolegend, San Diego, CA USA), CD38–R-phycoerythrin cyanine7 (CD38–PE-Cy™7; Biolegend), and CD96–R-phycoerythrin (CD96-PE; Biolegend) in Cell Staining Buffer (Biolegend); Annexin V–FITC and CD38–PE-Cy™7 were used for KG-1, KG-a, and TF-1 cells, and CD34–APC was also added for primary cells. The procedure was as follows: The cells (1 × 10^6^) were collected and resuspended in 100 μl Cell Staining Buffer, the antibody was added and incubated at 4 °C for 15 min, washed again with cell staining buffer one time, and then subject to the apoptosis assay. To exclude non-viable cells, cells were stained with 7AAD before flow cytometry (BD Versa, San Diego, CA, USA) analysis for antigen detecting.

### Real-time fluorescence quantitative polymerase chain reaction (qPCR)

Total RNA was extracted using TRIzol (Invitrogen, MA, USA) and immediately transcribed into cDNA by a Reverse Transcription Kit (Toyobo, Japan). Real-time qPCR was performed with a SYBR Green PCR Kit (Roche, Basel, Switzerland) on an ABI PRISM 7500 PCR instrument (Applied Biosystems). The reaction solution (20 μl) contained 10 μl 2 × QuantiTect SYBR Green PCR Master Mix, 0.5ul of each primers (10 nM), 2 μl of cDNA, and 7 μl of RNase-free water. Amplification was performed in three stages: holding stage (50 °C for 2 min and 95 °C for 10 min), cycling stage (40 cycles of 95 °C for 15 s, 60 °C for 1 min), and melting stage (95 °C for 15 s, 60 °C for 1 min, 95 °C for 30 s, 60 °C for 15 s). Relative gene expression levels were calculated using the 2-ΔΔCt method with β-actin as an internal control. Primer sequence listed in Additional file 10: Table S2.

### RNA sequencing analysis

KG-1 cells were dissolved in TRIzol after treatment with HHT, ATO, or their combination for 6 h, and then RNA was isolated. The sequencing reaction was performed using Illumina HiSeq 4000. Differentially expressed genes, cluster analysis, gene ontology (GO) enrichment analysis, pathway enrichment analysis, and other functional annotations were performed at Shanghai Aksomics Company Limited (Shanghai, China).

### Western blot analysis

KG-1 cells and primary CD34^+^ cells were collected and lysed in radioimmunoprecipitation assay lysis buffer (Beyotime, Shanghai, China) with phenylmethanesulfonyl fluoride, protease inhibitor cocktail, and phosphatase inhibitor cocktail (KangCheng Bio-tech, Shanghai, China) after treatment with drugs for 24 h. To ensure uniform sample amounts in each lane (adjusted to the homogeneous internal control from each sample), proteins were separated by 8% sodium dodecyl sulfate-polyacrylamide gel electrophoresis and then transferred to nitrocellulose membranes (Life Science, Germany). After blocking with 5% non-fat milk, the membranes were incubated overnight at 4 °C with the primary antibody (1:1000, all from Cell Signaling Technology, Beverly, MA, USA), followed by secondary horseradish peroxidase-conjugated antibody (1:5000, Cell Signaling Technology) in tris-buffered saline with Tween for 1 h (P53, antibodies for 2 h) at room temperature. Pathway signals were detected using an ECL kit (CWBIO, Beijing, China) and visualized on a ChemiDOC™ imaging system (Bio-Rad, Hercules, CA, USA). GAPDH was used as a loading control.

### Animal xenograft leukemia model

NRG mice (*Rag1*^−/−^, IL2R γ^−/−^ NOD mice) were purchased from the Nanjing Model Animal Center, and bred in a pathogen-free environment supplied with sterile food and water at the Experimental Animal Center of Fujian Medical University. All animal experimental protocols were approved by Fujian Medical University Animal Care and Use Committee. Adult mice (4–6 weeks old) were sublethally irradiated with 2 Gy of total body irradiation, and then injected with 7 × 10^6^ KG-1 cells 24 h later in 200 μl PBS via the tail vein. The mice were monitored weekly for the percentage of hCD45^+^ cells in the peripheral blood and were monitored daily for disease symptoms (ruffled coat, hunched back, weakness, and reduced motility) for one month. Along with hCD45^+^ cells > 1% in peripheral blood of mice, the mice were then assigned randomly to four groups (*n* = 5 per group) administered daily with: PBS (vehicle), HHT, ATO, or HHT and ATO in combination. The doses were calculated as follows. HHT: 1 mg/60 kg per day × 9.1 = 152 ng/g per day; ATO: 10 mg/60 kg per day × 9.1 = 1.52 μg/g per day. One week after drug administration, the mice were killed and infiltration of leukemia cells was evaluated by hematoxylin and eosin (H&E) staining, FCM and confocal laser-scanning.

### Fluorescence microscopy

The spleens and bone marrow were removed from the mice at the end of the experiment and fixed with 4% paraformaldehyde, embedded in paraffin, and cut into slices. After dewaxing, washing with water, antigen retrieval, and spontaneous fluorescence quenching, the tissues were blocked with 3% bovine serum albumin (for goat antibody) or 10% normal rabbit serum (for other antibodies) for 30 min at room temperature. After incubating with the primary antibody (anti-CD34 and anti-CD45; Goodbio Technology, Wuhan, anti-P53 and anti-NF-κB2, CST) and secondary antibody (Goodbio Technology), the tissue slices were counterstained with DAPI (C1005, Beyotime), and images were taken with a NIKON ECLIPSE CI confocal laser-scanning microscope (Japan).

### Statistical analysis

Group comparisons of parametric data were performed using analysis of variance; for data with a skewed distribution, the Jonckheere-Terpstra test was applied. Two-group comparisons were made with Student’s t-test. Significance was used as *p* < 0.05 and error bars represent SD. All statistical analyses were carried out with SPSS version 22.0.

## Results

HHT and ATO synergistically reduce cell viability in leukemia stem-like cells and CD34^+^ primary cells.

**Fig. 1:**
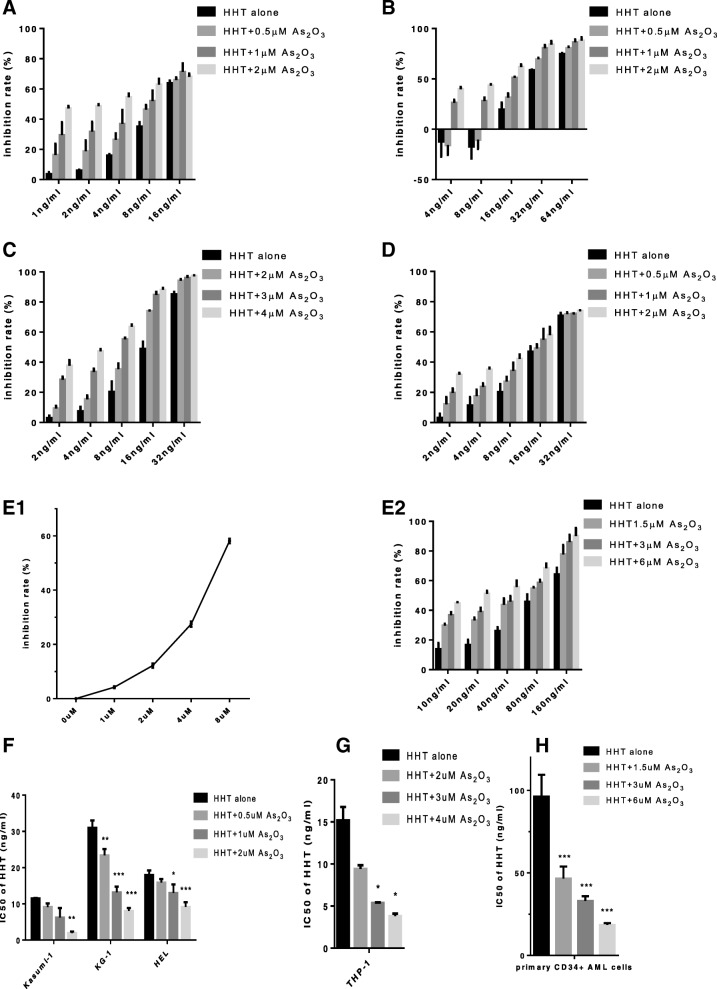
Arsenic trioxide (ATO) enhances the cytotoxic effect of homoharringtonine (HHT) in acute myeloid leukemia (AML) cell lines and primary CD34^+^ AML cells, and HHT and ATO synergistically damage leukemia stem-like cell lines and CD34^+^ primary cells (*n* = 3). Kasumi-1 cells (**a**), KG-1 cells (**b**), THP-1 cells (**c**), HEL cells (**d**), and primary CD34+ AML cells (**e1, e2**) were treated with HHT, ATO, or HHT + ATO for 2 days, and cell viability was measured by the CCK-8 assay. IC50 of HHT is calculated by using SPSS 22 (f,g,h). Error bars represent standard deviations of three independent experiments. *P* < 0.05*, *P* < 0.01**, *P* < 0.001***

Based on the results of the CCK-8 assay (data not shown), we selected the cell lines most sensitive to HHT and ATO treatment, Kasumi-1, KG-1, HEL, and THP-1, to evaluate the synergistic effect of the two reagents. Additional file 1: Figure S1 and Additional file 2: Figure S2 demonstrate that HHT and ATO reduced the viability of these four cell lines in time- and dose-dependent manners. Although ATO clearly boosted the cytotoxic effect of HHT on all cell lines (Fig. 1), synergistic effects were only detected for Kasumi-1 and KG-1 cells. These synergistic actions of HHT combined with ATO were also detected in CD34^+^ primary AML cells (*n* = 3) (Table 1), demonstrating that these effects are mainly exerted in LSCs. Next, we selected the ATO dose of maxim synergistic effects (bolded number in Table 1) as following experiments.

**+:** CI value

	Kasumi-1	KG-1	HEL	THP-1		CD34^+^ primary cells (*n* = 3)	
ATO (μM)	CI value			ATO (μM)	CI value	ATO (μM) CI value	
0.5	0.967	0.881	1.017	2	1.059	1.5	0.707
1	0.879	0.678	0.99	3	1.012	3	0.639
2	0.869	0.765	1.038	4	1.128	6	1.080

### ATO enhances the ability of HHT to induce cell apoptosis and cell cycle arrest in leukemia stem-like cells

**Fig. 2:**
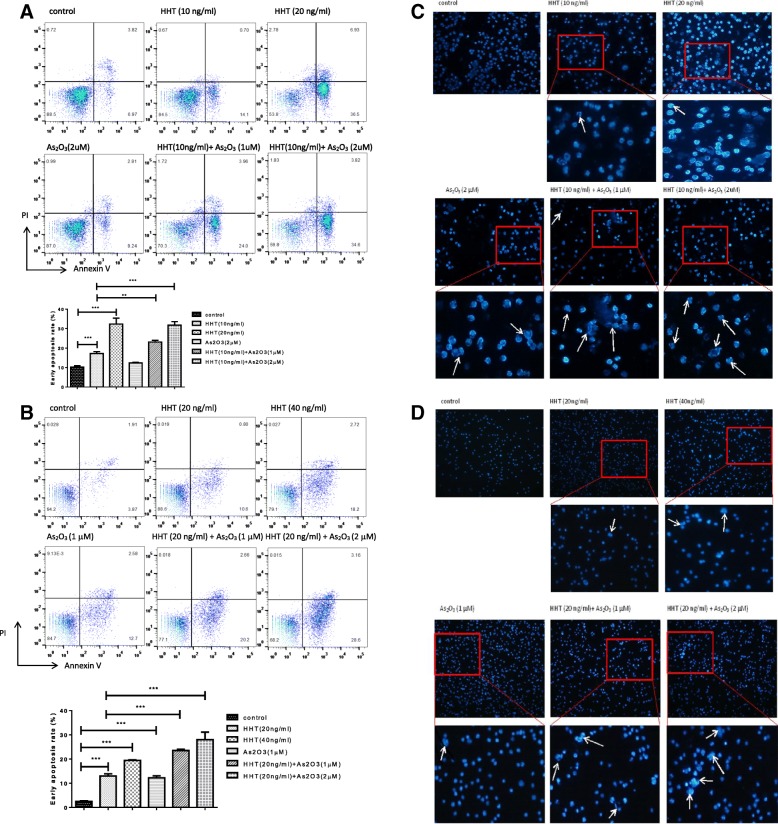
Arsenic trioxide (ATO) cooperates with homoharringtonine (HHT) to induce apoptosis in leukemia stem-like cell lines. Cells were treated with HHT, ATO, or HHT + ATO for 2 days, and apoptosis of Kasumi-1 (**a**) and KG-1 (**b**) cells was detected with PI/Annexin V and FACS. Error bars represent three independent experiments. *P* < 0.05*, *P* < 0.01**, *P* < 0.001***. Hochest dye was used to evaluate the shape of the nuclei of Kasumi-1 (**c**) and KG-1 (**d**) cells under fluorescence microscopy

To further identify the mechanism of damage induced to LSCs by HHT and ATO, we detected the apoptosis and cell cycle of Kasumi-1 and KG-1 cells after treatment. Flow cytometry showed that 10 ng/ml of HHT mildly induced the apoptosis of Kasumi-1 cells, and 2 μM ATO had only a slight apoptosis-inducing effect. However, their combination markedly increased the apoptosis rate of Kasumi-1 and KG-1 cells as well, exceeding the total of these two drugs independently (Fig. 2a, b). Moreover, compared with single-agent treatments, HHT + ATO caused increased nucleus fragmentation, as part of the nucleus began to dissolve (Fig. 2c, d).

The cell cycle was arrested in the G0/G1 phase in Kasumi-1 cells after HHT treatment, and this effect was also enhanced by ATO (Additional file 3: Figure S3A). Although the KG-1 cell cycle was also arrested in G0/G1 phase after HHT treatment, there was no further increase in the proportion of G0/G1-phase cells with the addition of ATO; instead, the combined treatment decreased the G2/M-phase cells (Additional file 3: Figure S3B). This suggested that ATO could strengthen the cytotoxic ability of HHT to G0/G1 phase cells, which are resistant to chemotherapy drugs.

### ATO enhances the effect of HHT in decreasing the proportion of LSCs

**Fig. 3:**
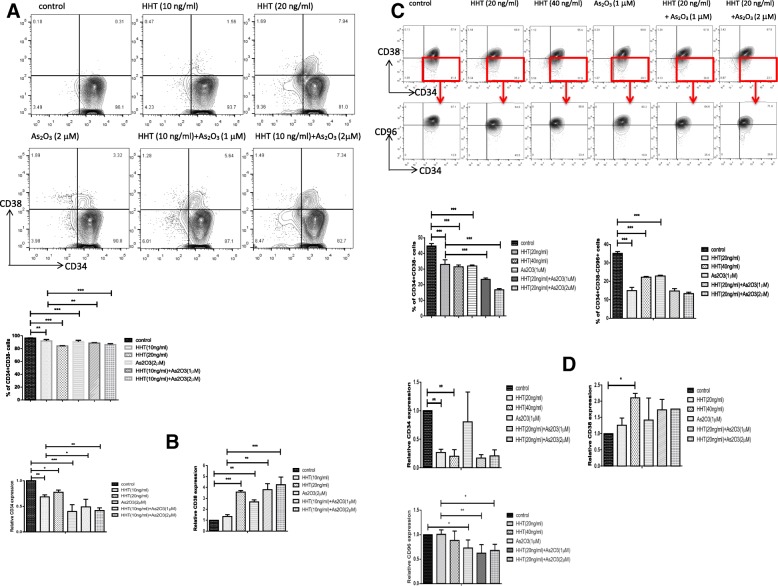
Arsenic trioxide (ATO) promotes the ability of homoharringtonine (HHT) to decrease the proportion of CD34^+^ CD38^−^ cells. Cells were treated with HHT, ATO, or HHT + ATO for 2 days, and cell surface antigen of Kasumi-1 (**a**) and KG-1 (**c**) cells was detected using FACS. The relative expression levels of *CD34, CD38*, and *CD96* mRNA of Kasumi-1 (**b**) and KG-1 (**d**) cells were quantified using qPCR. Error bars represent three independent experiments. *P* < 0.05*, *P* < 0.01**, *P* < 0.001***

Almost all of the Kasumi-1 cells were CD34^+^CD38^−^; however, they did not express the LSC marker CD96 (data not shown). After treatment with 10 ng/ml HHT, the proportion of CD34^+^CD38^−^ cells was slightly reduced, whereas 20 ng/ml HHT remarkably decreased the proportion of CD34^+^CD34^−^ cells. Moreover, when combining 10 ng/ml HHT with 1 μM or 2 μM ATO, the proportion of CD34^+^CD38^−^ cells dramatically declined (Fig. 3a). Similar results were found for KG-1 cells. Moreover, HHT also reduced the proportion of CD34^+^CD38^−^CD96^+^ cells in KG-1 cells, but increasing the concentration of HHT or adding ATO did not cause a further decrease (Fig. 3c). These results were confirmed by qPCR demonstrating downregulation in *CD34* expression and upregulation of *CD38* expression by HHT, and ATO promoted these effects, in both cell lines. In addition, *CD96* expression was also downregulated in KG-1 cells by HHT, and the effect was enhanced by the addition of ATO (Fig. 3b, d).

### HHT combined with ATO more effectively killed the CD34^+^CD38^−^ LSCs than CD34^+^CD38^+^ AML cells

**Fig. 4:**
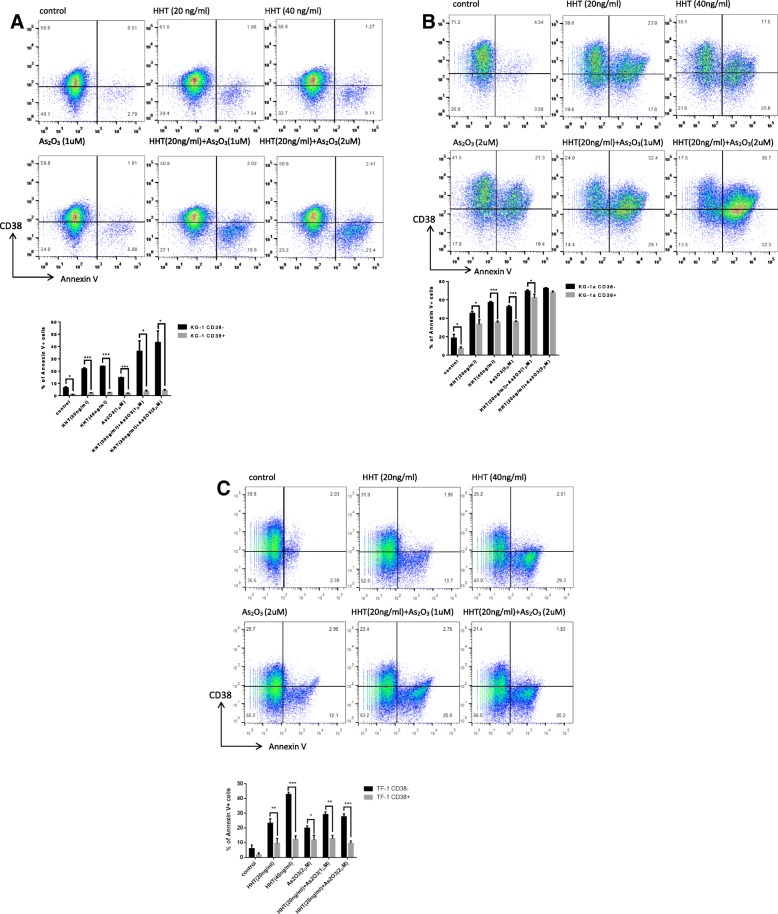
Homoharringtonine (HHT) combined with arsenic trioxide (ATO) more effectively killed the CD34^+^ CD38^−^ leukemia stem cells. Cells were treated with HHT, ATO, or HHT + ATO for 2 days, and then stained with CD38 antibody and Annexin V; the apoptosis rate of cells with different expression levels of CD38 was detected using FACS. Error bars represent three independent experiments. *P* < 0.05*, *P* < 0.01**, *P* < 0.001***

To further explore the mechanism for the decreased proportion of CD34^+^CD38^−^ cells in KG-1 and Kasumi-1 cells, we examined the effect of HHT on cell differentiation of KG-1; however, no differentiation induction was observed (data not shown). To further clarify the mechanism, we used double staining of Annexin V and anti-CD38, which showed that (after excluding dead cells), Annxein V-positive cells mainly assembled in the section of CD38-low KG-1 cells (Fig. 4a). The same result was found in two other two cell lines, KG-1a (Fig. 4b) and TF-1 (Fig. 4c), with similar CD38 expression to KG-1 cells. To further tease apart the effects of apoptosis, which would reduce the contents of all proteins, and low CD38 expression, we analyzed the CD38^high^ and CD38^low^ KG-1 and TF-1 (CD34^+^) cells separately. Consistently, flow cytometry showed that the combined HHT and ATO treatment more effectively killed the CD34^+^CD38^−^ LSCs (Additional file 4: Figure S4).

### ATO promotes the ability of HHT to damage primary LSCs in serum-free medium with cytokines

**Fig. 5:**
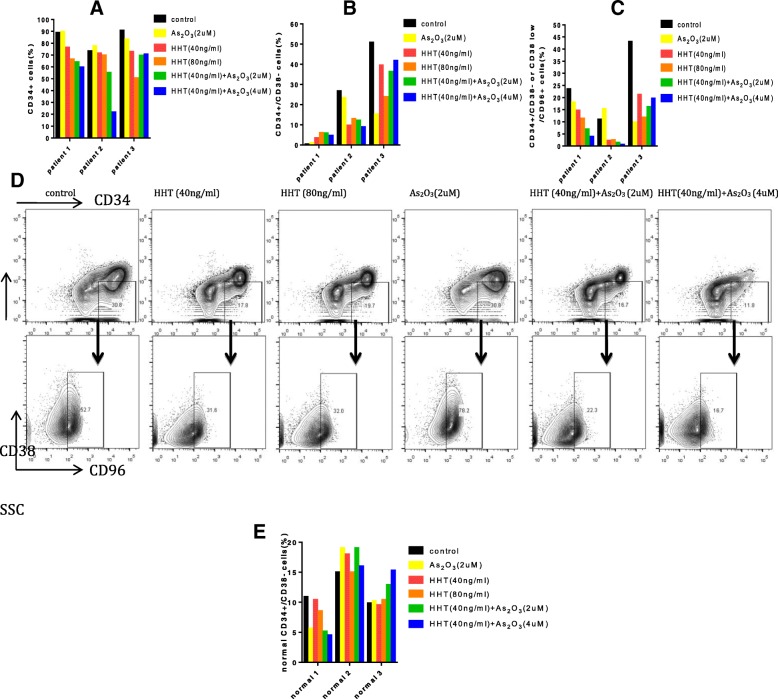
Homoharringtonine (HHT) combined with arsenic trioxide (ATO) decrease the proportion of primary leukemia stem cells (LSCs) in serum free medium with cytokine cocktail (Flt3L, SCF, IL-3 and IL-6). Quantification of frequencies of CD34^+^cells (**a**), CD34^+^/CD38^−^ cells (**b**) and CD34^+^/CD38^−^/CD96^+^ cells (**c**). (**d**) Display of flow cytometric analysis on bone marrow sample of patient no. 2 after treatment with HHT and ATO alone or combined. (**e**) Represents the proportion of normal primary CD34+/CD38- (*n* = 3)

To generalize the above findings in LSCs for more complex and universal systems, we treated bone marrow mononuclear cells from AML patients (*n* = 4) with the two drugs. To simulate the survival condition of primary LSCs, we incubated the primary cells with the drugs in serum-free medium with Flt3L, SCF, IL-3, and IL-6. Similar to the results of the cell lines (KG-1, KG-1a, TF-1), the proportions of CD34^+^ cells, CD34^+^/CD38^−^ cells, and CD34^+^/CD38^−^/CD96^+^ cells decreased after treating with HHT, with a more dramatic reduction observed in the combined treatment group (Fig. 5a–d, Additional file 5: Figure S5). However, the proportions of normal CD34+/CD38- cells (*n* = 3) have no observably decrease (Fig. 5e).

Moreover, HHT more effectively damaged the CD34^+^/CD38^−^ primary AML cells than CD34^+^/CD38^−^ cells, and ATO significantly promoted this effect in spite of a lack of an obvious influence on the apoptosis rate of these cells when treated alone (Additional file 6: Figure S6).

### HHT upregulates the notch pathway while ATO downregulates the NF-κB pathway

High throughput mRNA-sequencing analysis demonstrated that HHT upregulates the Notch and NF-κB pathway, as well as P53 family members (Additional file 7: Figure S7), suggesting the subsequent inhibition of proliferation in AML cells, at least partly, followed by P53 upregulation and BCL-2 downregulation [9, 12]. In addition, ATO could inhibit the NF-κB pathway in MCL (mantle cell lymphoma) [15]. We also found that ATO downregulated the NF-κB pathway from sequencing analysis data. Thus, we hypothesized that HHT activates the Notch pathway and P53 to kill LSCs, while ATO inhibits the NF-κB pathway to produce the observed synergistic effect.

### HHT activates the P53 and NF-κB pathways, and ATO strongly inhibits the NF-κB pathway

**Fig. 6:**
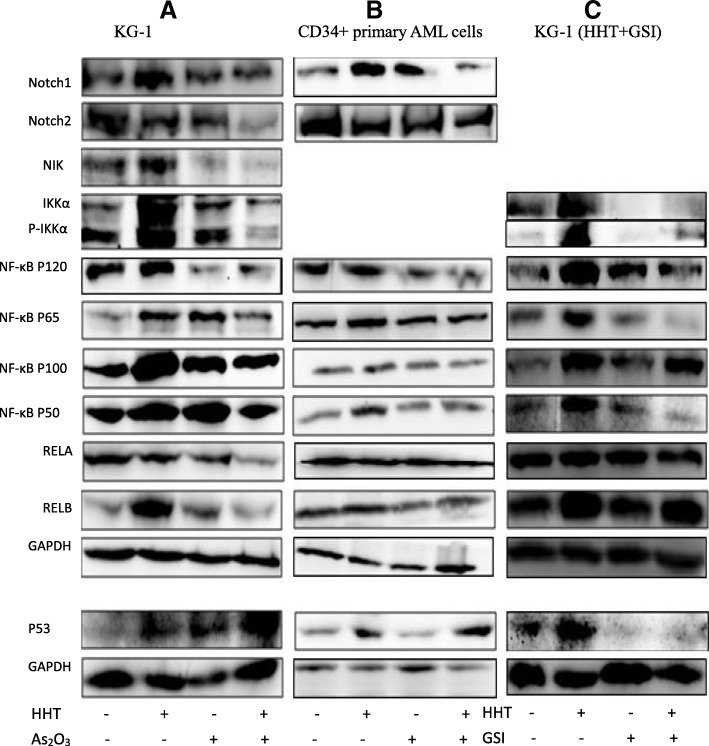
Western bloting analysis for protein expression. KG-1 (**a**) and CD34^+^ primary cells (**b**) were treated with HHT, arsenic trioxide (ATO) alone or in combination for 24 h, and total proteins were separated for western blotting. (**c**) GSI (1µM) eliminated the influence of homoharringtonine HHT to Notch, NF-κB, and P53

To confirm the RNA-sequencing result and test our hypothesis, the effects of the treatments on the relevant protein levels were assessed with western blotting. The HHT (20 ng/ml)-induced upregulation of Notch1 and NF-κB was reversed when combined with ATO (1 μM) in KG-1 cells (Fig. 6a). These effects were further confirmed by the fact that P53 was activated by both HHT and ATO, which was also observed in primary CD34^+^ cells (Fig. 6b). Further, treatment of gamma secretase inhibitor (GSI)-LY3039478, Notch1 inhibitor to the KG-1 cells incubated with HHT resulted in inhibition of most of the proteins involved in the p53, NIK/IKKα, and NF-κB pathways (Fig. 6c).

### HHT and ATO reduce the xenograft burden and proportion of LSCs in the spleen rather than the bone marrow

**Fig. 7:**
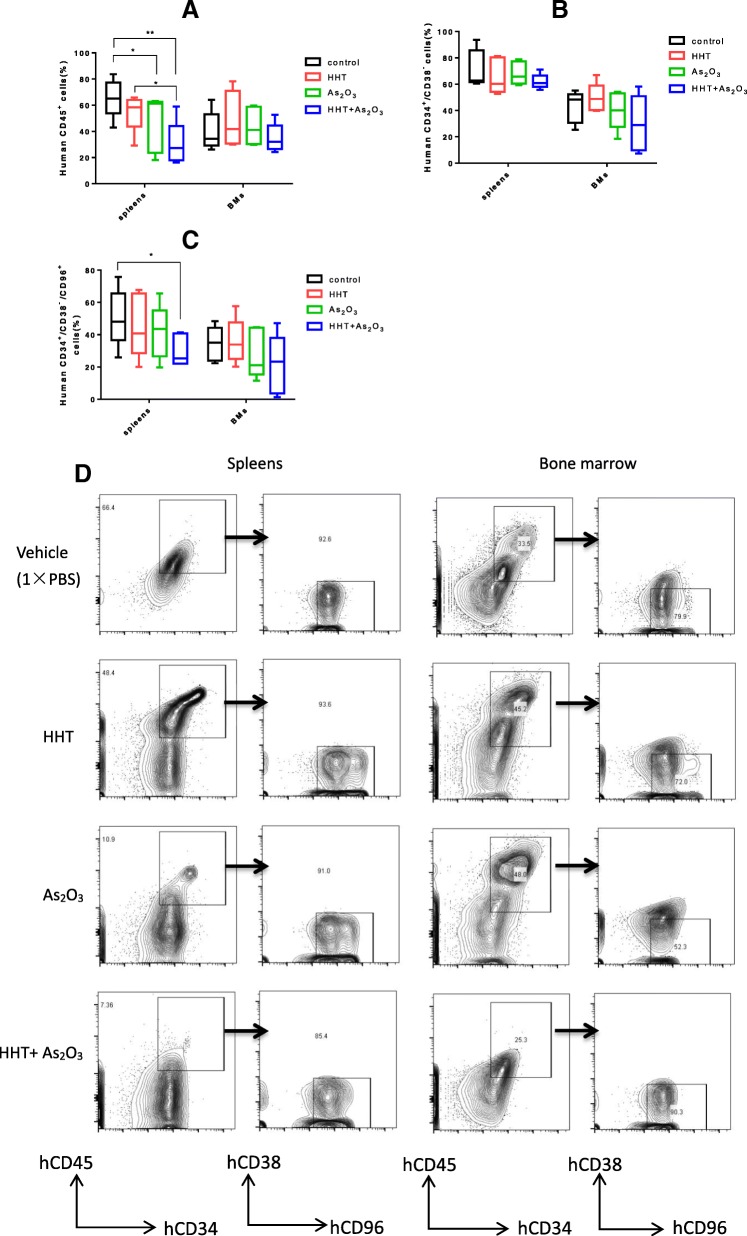
Homoharringtonine (HHT) combined with arsenic trioxide (ATO) efficaciously eliminated the leukemia stem cells (LSCs) in a xenograft model of NRG mice (*n* = 5). (**a**) Evaluation of enrichment of KG-1 cells in the spleens and bone marrow of mice by analysis of hCD45 expression using flow cytometry. (**b**, **c**) Detection the residual of LSCs (CD34^+^CD38^−^CD96^+^) in the spleens and bone marrow using flow cytometry. (**d**) Representative flow cytometric analysis of stem LSCs markers (CD34, CD38, CD96). *P* < 0.05*, *P* < 0.01**, *P* < 0.001***

**Fig. 8:**
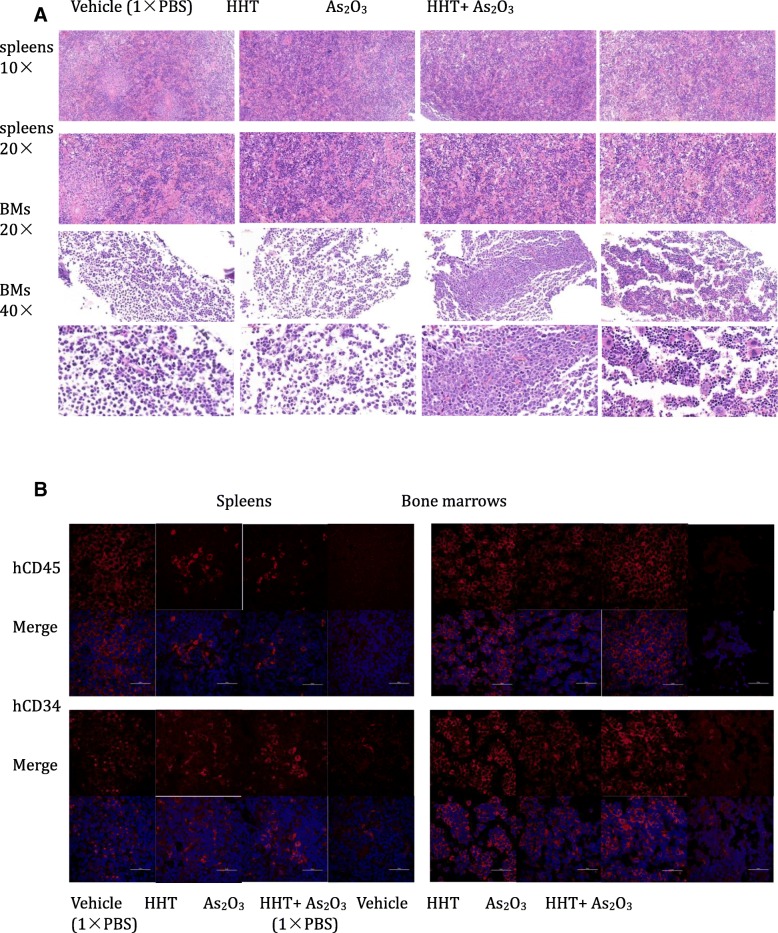
Homoharringtonine (HHT) combined with arsenic trioxide (ATO) remarkably obliterated the histological infiltration of leukemia stem cells (LSCs). (**a**) H&E-stained sections of representative 4% paraformaldehyde-fixed spleens and bone marrow from NRG mice. (**b**) hCD45 and hCD34 levels were detected in the different groups by confocal laser-scanning microscopy in representative 4% paraformaldehyde-fixed spleens and bone marrow samples from NRG mice. Scale bars: 50 μm

The burden of human leukemia cells (KG-1 cells) in the spleen of NRG mice was markedly reduced with combined administration of HHT and ATO (*P* < 0.01). Moreover, ATO enhanced the action of HHT (HHT vs. HHT + ATO, *P* < 0.05), although there was no significant effect with HHT administration alone (Fig. 7a, d). However, this treatment did not appear to impact the leukemia cells in the bone marrow of mice (Fig. 7a). Further, LSCs were effectively obliterated with the combined drug treatment (*P* < 0.05), but not with either treatment alone (Fig. 7c), although there was no elimination effect in the bone marrow. Moreover, histological analysis demonstrated that the ability of KG-1 cells to invade the spleen was remarkably impaired in mice treated with HHT and ATO compared with that observed in mice that received vehicle as a control. Again, this effect was not apparent in the bone marrow (Fig. 8a). Confocal laser-scanning microscopy clearly showed that the expression levels of hCD45 (leukemia cell marker) and hCD34 (a LSC marker) were significantly decreased in the combined treatment group compared with those of the other three groups (Fig. 8b). The expression of P53 and NF-κB2 in vivo were also evaluate by confocal laser-scanning. Like the result derived from WB, HHT and ATO could upregulated the expression of P53 and ATO reduce the expression of NF-κB2 which upregulated by HHT in spleens, it is not obvious in BM (Additional file 8: Figure S8).

## Discussion

The main clinical challenge in leukemia treatment remains the effective elimination of LSCs to reduce the chance of remission. Here, we show that HHT and ATO have a synergic effect to inhibit the proliferation of LSCs, which was identified in KG-1, Kasumi-1, primary CD34^+^ cells from AML patients, and in an in vivo xenograft model. This synergistic effect was found to involve greater induction of cell apoptosis and cell cycle arrest.

Shen et al. [18] pointed out that HHT may be an effective killing agent of LSCs in vitro, and decreased the ratio of CD34^+^CD38^−^CD96 ^+^ cells in KG-1 cells. Here, we expand these findings by demonstrating that HHT and ATO synergistically reduced the proportion of LSCs in the Kasumi-1 and KG-1 cells, as well as in primary bone marrow cells. Moreover, we identified the underlying mechanism. However, the reason for the decreasing proportion of LSCs was unknown. At first, despite qPCR results indicating that ATO enhanced the effects of HHT in reducing the expression of levels *CD34* and *CD96* mRNA and upregulating *CD38* expression, cell differentiation was not observed. Thus, we speculated that the mechanism is not underlying in molecule expression regulation. Although a previous study [19] demonstrated that HHT had a greater potency to kill the CD34^+^CD38^−^ primary AML cells compared to CD34^+^CD38^+^ cells, the sample size was limited. Our results further confirm these findings with a larger sample size along with confirmation in other cell lines. Apoptotic cells (Annexin V^+^ cells) largely localized in the section of CD38^−^ or CD38^low^ cells in all three cell lines; namely, HHT or HHT combined with ATO more effectively killed CD34^+^CD38^−^ KG-1, KG-1a, TF-1 cells than CD34^+^/CD38^+^ cells. We further validated the decrease of primary CD34^+^/CD38^−^/CD96^+^ cells and a higher apoptosis rate of primary CD34^+^/CD38^−^ or CD38^low^ cells than CD34^+^/CD38^+^ cells in bone marrow cells after treatment with HHT and ATO. These findings were confirmed in CD38^+^ and CD38^−^ KG-1 and TF-1 cells analyzed separately, demonstrating that HHT does not simply inhibit the expression of all proteins through apoptosis induction [20]. However, it remains unclear why CD34^+^/CD38^−^ cells are more sensitive to HHT and HHT combined with ATO.

Chen et al. [8] first uncovered that the synergistic effect of HHT and ATO involved upregulation of the PI3k/Akt pathway by ATO in U937 cells. We found only a slight effect of ATO alone, although it markedly enhanced the actions of HHT on LSCs at the same dose. This suggested further synergic mechanisms, which were identified as regulation of the Notch, NF-kB, and P53 pathways.

Notch is considered to be an oncogene in T-cell acute lymphoblastic leukemia and lymphoma [21, 22]; however, its role in AML has been controversial [12, 23–25]. The majority of related studies support that Notch acts as an inhibitor in AML. The Notch pathway also interacts with stemness-related pathways, including the Wnt or Hedgehog signaling pathway [9]. Here, we found that HHT activates the Notch pathway, leading to upregulation of the NF-κB pathway and P53, whereas ATO not only inhibited the NF-κB pathway but also further induced the expression of P53. We believe that this is, at least, part of the mechanism underlying the synergic effect of these two drugs on LSCs. In addition, HHT upregulated Notch1 and downregulated Notch2, confirming a previous study showing that Notch2 knockdown increased the level of Notch1 expression in THP-1 and TMD7 cell lines [26]. However, the mechanism and biological significance of the influence of HHT on Notch1 and Notch2 are unclear and worthy of further study.

Interestingly, in the xenograft model, the leukemia cell burden and LSCs reduction was more remarkable in the spleen with no significant difference in the bone marrow. This may be related to a low sample size or the effect of bone marrow niche. However, the specific mechanism of action of these two drugs in vivo should be further evaluated toward promoting their clinical application and elucidating the mechanism to improve treatment strategies for AML patients.

## Conclusions

Overall, our results demonstrate a clear synergistic effect of HHT and ATO on LSCs in vitro and in vivo. We suggest that the chemotherapy regimens including HHT and ATO could decrease the rate of AML relapse.

## Additional files

Additional file 1: **Figure S1.** Homoharringtonine (HHT) reduced the cell viability of acute myeloid leukemia (AML) cell lines. Kasumi-1 cells (A), KG-1 cells (B), THP-1 cells (C), and HEL cells (D) were treated with different concentrations of HHT for 12 h, 24 h, and 48 h, and cell viability was measured by the CCK-8 assay. Error bars represent standard deviations of three independent experiments. (DOCX 301 kb)

Additional file 2: **Figure S2.** Arsenic trioxide (ATO) decreased the cell viability in acute myeloid leukemia (AML) cell lines. Kasumi-1 cells (A), KG-1 cells (B), THP-1 cells (C), and HEL cells (D) were treated with different concentrations of ATO for 1, 2, and 3 days, and cell viability was measured by the CCK-8 assay. Error bars represent the standard deviations of three independent experiments. (DOCX 176 kb)

Additional file 3: **Figure S3.** Arsenic trioxide (ATO) cooperates with homoharringtonine (HHT) to arrest the cell cycle in leukemia stem-like cell lines. Cells were treated with HHT, ATO, or HHT + ATO for 2 days, and DNA contents of Kasumi-1 (A) and KG-1 (B) cells were detected with PI/RNAase and FACS. Error bars represent three independent experiments. (DOCX 154 kb)

Additional file 4: **Figure S4.** Homoharringtonine (HHT) combined with arsenic trioxide (ATO) more effectively killed the CD34^+^CD38^−^ leukemia stem cells sorted from KG-1 and TF-1 cells. Cells were sorted by FACS Aria II according to the expression of CD38. CD38^high^ or CD38^low^ cells were treated with HHT, ATO, or HHT + ATO for 2 days, and then stained with Annexin V; the apoptosis rate of the cells was detected using FACS. Error bars represent three independent experiments. *P* < 0.05*, *P* < 0.01**, *P* < 0.001***. (DOCX 527 kb)

Additional file 5: **Figure S5.** Homoharringtonine (HHT) combined with arsenic trioxide (ATO) decrease the proportion of primary leukemia stem cells (LSCs) in serum free medium with cytokine cocktail (Flt3L, SCF, IL-3 and IL-6). Quantification of frequencies of CD34^+^cells (A), CD34^+^/CD38^−^ cells (B) and CD34^+^/CD38^−^/CD96^+^ cells (C) from patient 4. (D) Display of flow cytometric analysis on bone marrow sample after treatment with HHT and ATO alone or combined. (DOCX 189 kb)

Additional file 6: **Figure S6.** Homoharringtonine (HHT) combined with arsenic trioxide (ATO) more effectively damaged the primary CD34^+^CD38^−^ cells than CD34^+^/CD38^+^ cells in serum-free medium with a cytokines cocktail (Flt3L, SCF, IL-3 and IL-6). (A–C) Quantification of frequencies of Annexin V-positive cells in CD34^+^CD38^−^ and CD34^+^CD38+ cells from patient 1 (A), patient 2 (B), patient 3 (C), patient 4 (D). (E) Representative flow cytometric analysis of patient 2 for apoptosis using Annexin V and stem cells markers (CD34, CD38). (DOCX 376 kb)

Additional file 7: **Figure S7.** RNA sequencing and functional enrichment analysis. KG-1 cells were treated with Homoharringtonine (HHT) and arsenic trioxide (ATO) alone or combined for 6 h and then RNA was isolated, after mRNA sequencing performed, cluster analysis and pathway enrichment analysis were applied. (DOCX 224 kb)

Additional file 8: **Figure S8.** Homoharringtonine (HHT) combined with arsenic trioxide (ATO) alter the expression of P53 and NF-κB2. P53 and NF-κB2 levels were detected in the different groups by confocal laser-scanning microscopy in representative 4% paraformaldehyde-fixed spleens and bone marrow samples from NRG mice. Scale bars: 50 μm (DOCX 1214 kb)

Additional file 9: **Table S1.** Patients characteristic (DOCX 27 kb)

Additional file 10: **Table S2.** Primer Sequences for PCR (DOCX 18 kb)

## Data Availability

The datasets used and/or analysed during the current study are available. from the corresponding author on reasonable request.

## Abbreviations

ATO: arsenic trioxide
FITC: fluorescein isothiocyanate
HHT: homoharringtonine
LSCs: leukemia stem cells
NF-κB: nuclear factor-kappa B
PBS: phosphate buffered saline
PE: phycoerythrin
PE-Cy7: phycoerythrin cyanine 7
PI: propidium iodide
