# Correction to: Synergistic killing effects of homoharringtonine and arsenic trioxide on acute myeloid leukemia stem cells and the underlying mechanisms

**DOI:** 10.1186/s13046-019-1377-7

**Published:** 2019-09-16

**Authors:** Ming Tan, Qian Zhang, Xiaohong Yuan, Yuanzhong Chen, Yong Wu

**Affiliations:** 1 Fujian Institute of Hematology, Fujian Provincial Key Laboratory on Hematology, Fujian Medical University Union Hospital, 29 Xinquan Road, Fuzhou, 350001 Fujian China; 2 Fujian Medical University graduate school, 1 Xuefu North Road, Fuzhou, 350112 Fujian China

**Correction to: J Exp Clin Cancer Res**

**https://doi.org/10.1186/s13046-019-1295-8**

In the publication of this article [1], there are two corrections: 1. The corresponding author Yuanzhong Chen’s email should be changed to chenyz@mail.fjmu.edu.cn; 2. Several figures Fig. 5, Fig. 8, and Additional files 5, 6, 9 and 10 need to be corrected, because the formats are wrong, and the revised figures are shown below.

**Fig. 5:**
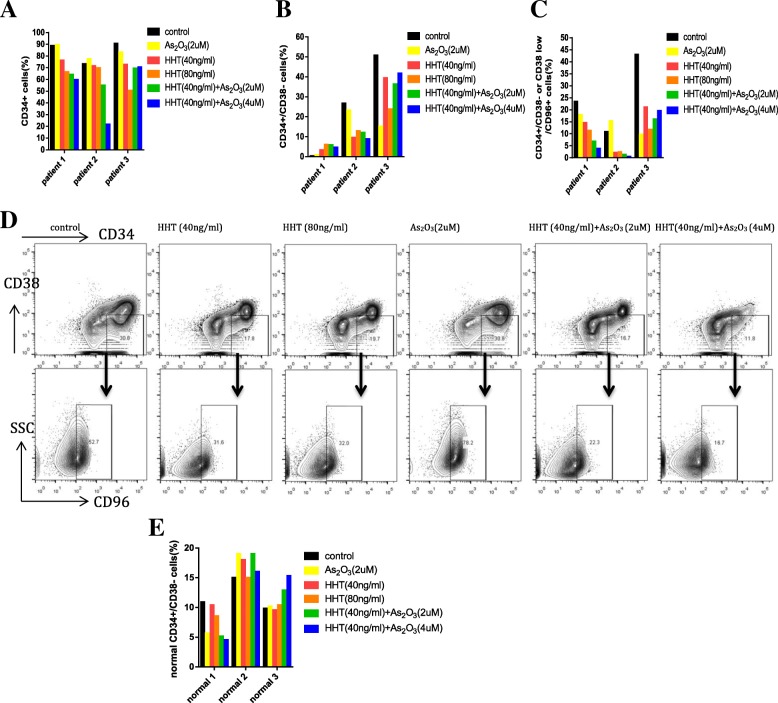
Homoharringtonine (HHT) combined with arsenic trioxide (ATO) decrease the proportion of primary leukemia stem cells (LSCs) in serum free medium with cytokine cocktail (Flt3L, SCF, IL-3 and IL-6). Quantification of frequencies of CD34^+^cells (**a**), CD34^+^/CD38^−^ cells (**b**) and CD34^+^/CD38^−^/CD96^+^ cells (**c**). (**d**) Display of flow cytometric analysis on bone marrow sample of patient no. 2 after treatment with HHT and ATO alone or combined. (**e**) Represents the proportion of normal primary CD34+/CD38- (*n* = 3)

**Fig. 8:**
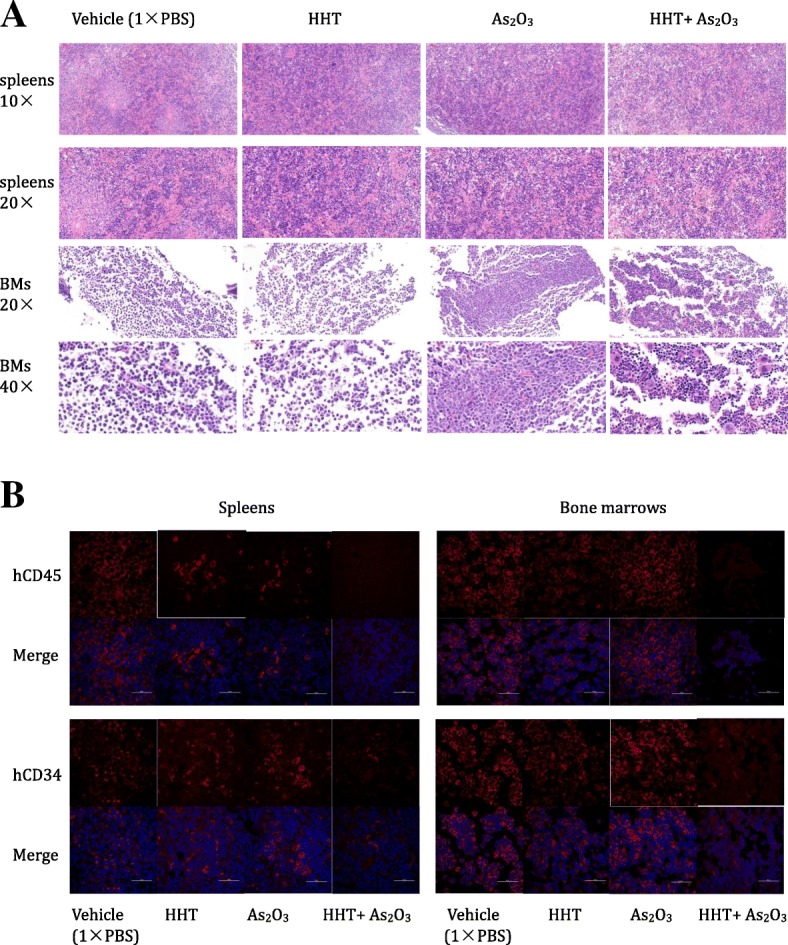
Homoharringtonine (HHT) combined with arsenic trioxide (ATO) remarkably obliterated the histological infiltration of leukemia stem cells (LSCs). (**a**) H&E-stained sections of representative 4% paraformaldehyde-fixed spleens and bone marrow from NRG mice. (**b**) hCD45 and hCD34 levels were detected in the different groups by confocal laser-scanning microscopy in representative 4% paraformaldehyde-fixed spleens and bone marrow samples from NRG mice. Scale bars: 50 μm

**Table S1:** Patients characteristic

NO.	Gender	Age	WBC(*10^9^/L)	Hb (g/L)	PLT (*10^9^/L)	FAB type	BM Blasts(%)	Immune markers	Karyotype
1	female	23	1.85	124	168	M0	63.2	CD7, CD117, HLA-DR	46 XX
2	male	29	12.72	138	7	M5	60.3	CD7,HLA-DR, CD33	46 XY
3	male	32	1.14	46	4	M2a	80	MPO,CD99,CD117	46 XY t(8;21)
4	female	36	5.57	50	83	M5b	78	CD117, CD33,MPO	46 XX
5	female	43	20.57	45	10	M1	72	CD117, CD33, MPO	46 XX
6	female	25	19.6	34	23	M0	69.3	CD7, CD117, MPO	46 XX
7	male	29	27	23	3	M5	59.6	CD33, HLA-DR CD15	46 XY

NO.1-4 were used to FCM (Flow Cytometry) analysis; NO.5-7 were used to synergistic effect. NO.7 were also used for WB

**Table S2:** Primer Sequences for PCR

Gene	Primer Sequences
β-actin	Forward 5’-GCCAACCGCGAGAAGATGA-3’
	Reverse 5’-CATCAGGATGCCAGTGGT-3’
CD34	Forward 5’- ACTCGGTGCGTCTCTCTAGG -3’
	Reverse 5’- CCGTGAGACTCTGCTCTGC-3’
CD38	Forward 5’- TTG GGA ACTCAG ACC GTA CCT TG-3’
	Reverse 5’- CCA CAC CAT GTGAGG TCA TC-3’
CD96	Forward 5’- ACCACAGTCAAGGTTTTTG-3’
	Reverse 5’- CCAGGCTGGAGAAGGTTGG-3’

The original article has been corrected.

## Additional files

Additional file 5: Figure S5. Homoharringtonine (HHT) combined with arsenic trioxide (ATO) decrease the proportion of primary leukemia stem cells (LSCs) in serum free medium with cytokine cocktail (Flt3L, SCF, IL-3 and IL-6). Quantification of frequencies of CD34+cells (A), CD34+/CD38−cells (B) and CD34+/CD38−/CD96+ cells (C) from patient 4. (D) Display of flow cytometric analysis on bone marrow sample after treatment with HHT and ATO alone or combined.

Additional file 6: Figure S6. Homoharringtonine (HHT) combined with arsenic trioxide (ATO) more effectively damaged the primary CD34+CD38− cells than CD34+/CD38+ cells in serum-free medium with a cytokines cocktail (Flt3L, SCF, IL-3 and IL-6). (A–C) Quantification of frequencies of Annexin V-positive cells in CD34+CD38− and CD34+CD38+ cells from patient 1 (A), patient 2 (B), patient 3 (C), patient 4 (D). (E) Representative flow cytometric analysis of patient 2 for apoptosis using Annexin V and stem cells markers (CD34, CD38).

Additional file 9: Table S1. Patients characteristic.

Additional file 10: Table S2. Primer Sequences for PCR.
