# Supplementary figures and images for: Synergistic killing effects of homoharringtonine and arsenic trioxide on acute myeloid leukemia stem cells and the underlying mechanisms
Source: J Exp Clin Cancer Res. 2019 Jul 15;38:308. doi: 10.1186/s13046-019-1295-8 (PMC6631946; doi:10.1186/s13046-019-1295-8)

D

C

B

A

**Fig.S1.**


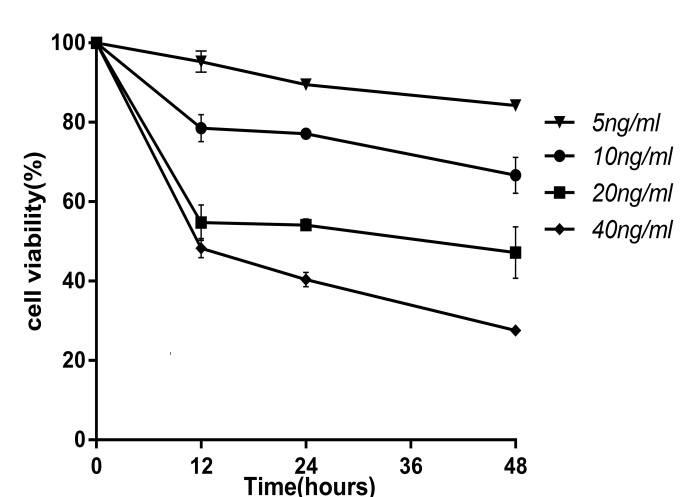

Supplement: Supplementary file 1 — Figure S1. Homoharringtonine (HHT) reduced the cell viability of acute myeloid leukemia (AML) cell lines. Kasumi-1 cells (A), KG-1 cells (B), THP-1 cells (C), and HEL cells (D) were treated with different concentrations of HHT for 12 h, 24 h, and 48 h, and cell viability was measured by the CCK-8 assay. Error bars represent standard deviations of three independent experiments. (DOCX 301 kb) [file 13046_2019_1295_MOESM1_ESM.docx]

**
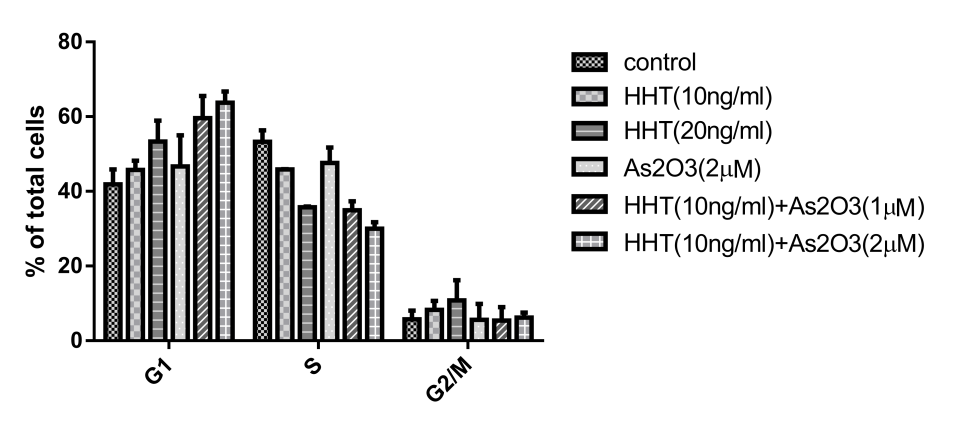
**

A

B

**
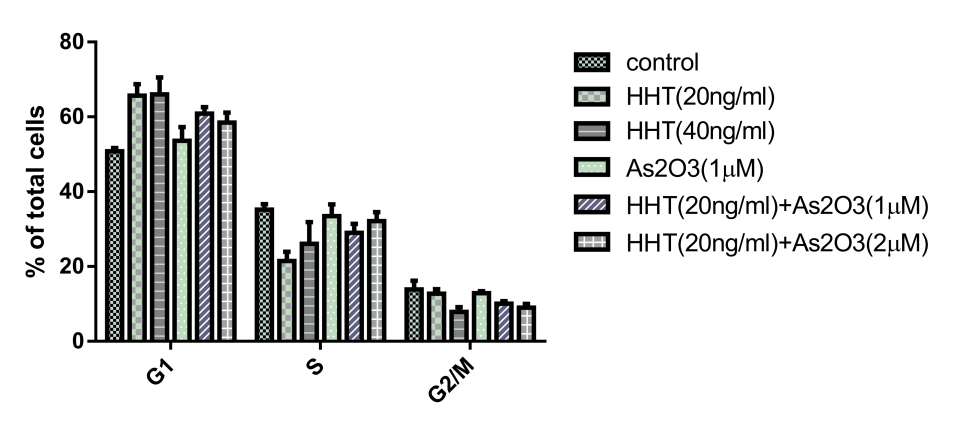
**

**Fig.S3**

Supplement: Supplementary file 3 — Figure S3. Arsenic trioxide (ATO) cooperates with homoharringtonine (HHT) to arrest the cell cycle in leukemia stem-like cell lines. Cells were treated with HHT, ATO, or HHT + ATO for 2 days, and DNA contents of Kasumi-1 (A) and KG-1 (B) cells were detected with PI/RNAase and FACS. Error bars represent three independent experiments. (DOCX 154 kb) [file 13046_2019_1295_MOESM3_ESM.docx]

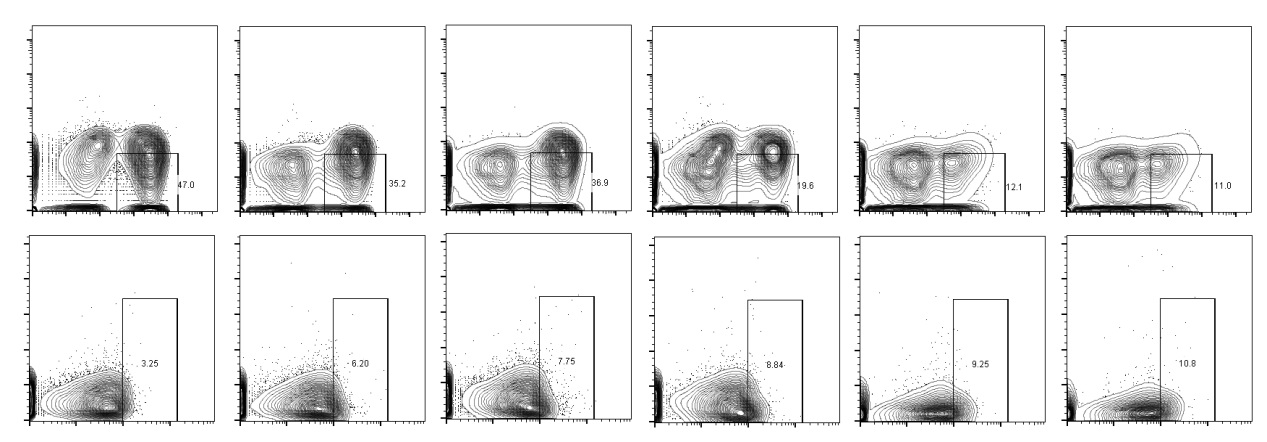


**Fig S5.**

A

B

C

D

CD38

SSC

CD96

CD34

control HHT (40ng/ml) HHT (80ng/ml) As2O3(2uM) HHT (40ng/ml) HHT(40ng/ml)

+As2O3 (2uM) +As2O3 (4uM)

Supplement: Supplementary file 5 — Figure S5. Homoharringtonine (HHT) combined with arsenic trioxide (ATO) decrease the proportion of primary leukemia stem cells (LSCs) in serum free medium with cytokine cocktail (Flt3L, SCF, IL-3 and IL-6). Quantification of frequencies of CD34+cells (A), CD34+/CD38− cells (B) and CD34+/CD38−/CD96+ cells (C) from patient 4. (D) Display of flow cytometric analysis on bone marrow sample after treatment with HHT and ATO alone or combined. (DOCX 189 kb) [file 13046_2019_1295_MOESM5_ESM.docx]

B

A

D

C

**
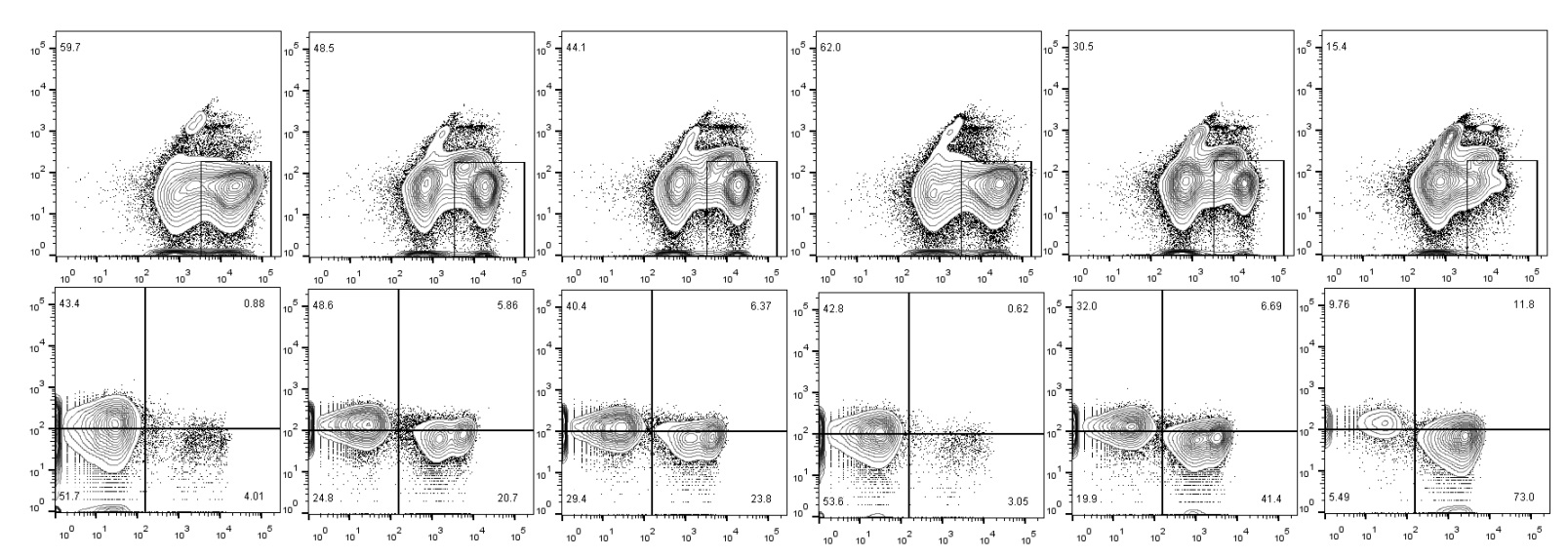
**

C

**Fig. S6.**

E

control HHT (40 ng/ml) HHT (80 ng/ml) As2O3 (2 μM) HHT (40 ng/ml)+As2O3 (2 μM) HHT (40n g/ml)+As2O3 (4 μM)

CD34

CD38

7-AAD

Annexin V

Supplement: Supplementary file 6 — Figure S6. Homoharringtonine (HHT) combined with arsenic trioxide (ATO) more effectively damaged the primary CD34+CD38− cells than CD34+/CD38+ cells in serum-free medium with a cytokines cocktail (Flt3L, SCF, IL-3 and IL-6). (A–C) Quantification of frequencies of Annexin V-positive cells in CD34+CD38− and CD34+CD38+ cells from patient 1 (A), patient 2 (B), patient 3 (C), patient 4 (D). (E) Representative flow cytometric analysis of patient 2 for apoptosis using Annexin V and stem cells markers (CD34, CD38). (DOCX 376 kb) [file 13046_2019_1295_MOESM6_ESM.docx]
