# Supplementary material for: Synergistic killing effects of homoharringtonine and arsenic trioxide on acute myeloid leukemia stem cells and the underlying mechanisms
Source: J Exp Clin Cancer Res. 2019 Jul 15;38:308. doi: 10.1186/s13046-019-1295-8 (PMC6631946; doi:10.1186/s13046-019-1295-8)
Supplement: Supplementary file 2 — Figure S2. Arsenic trioxide (ATO) decreased the cell viability in acute myeloid leukemia (AML) cell lines. Kasumi-1 cells (A), KG-1 cells (B), THP-1 cells (C), and HEL cells (D) were treated with different concentrations of ATO for 1, 2, and 3 days, and cell viability was measured by the CCK-8 assay. Error bars represent the standard deviations of three independent experiments. (DOCX 176 kb) [file 13046_2019_1295_MOESM2_ESM.docx]

D

A

B

C

**Fig. S2.**
