# Supplementary material for: Synergistic killing effects of homoharringtonine and arsenic trioxide on acute myeloid leukemia stem cells and the underlying mechanisms
Source: J Exp Clin Cancer Res. 2019 Jul 15;38:308. doi: 10.1186/s13046-019-1295-8 (PMC6631946; doi:10.1186/s13046-019-1295-8)
Supplement: Supplementary file 4 — Figure S4. Homoharringtonine (HHT) combined with arsenic trioxide (ATO) more effectively killed the CD34+CD38− leukemia stem cells sorted from KG-1 and TF-1 cells. Cells were sorted by FACS Aria II according to the expression of CD38. CD38high or CD38low cells were treated with HHT, ATO, or HHT + ATO for 2 days, and then stained with Annexin V; the apoptosis rate of the cells was detected using FACS. Error bars represent three independent experiments. P < 0.05*, P < 0.01**, P < 0.001***. (DOCX 527 kb) [file 13046_2019_1295_MOESM4_ESM.docx]

**
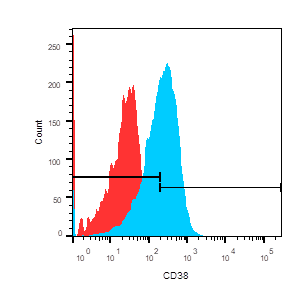

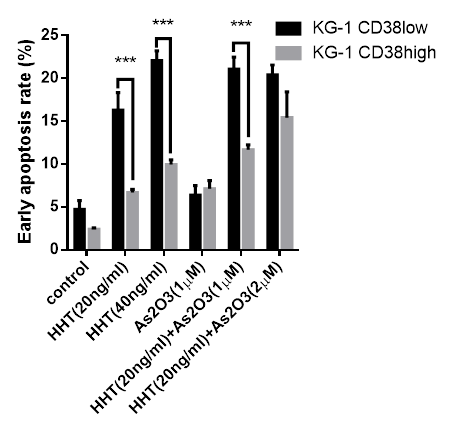

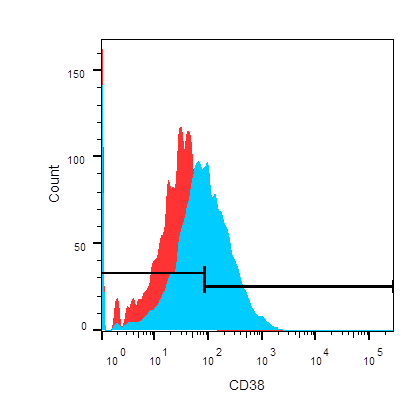

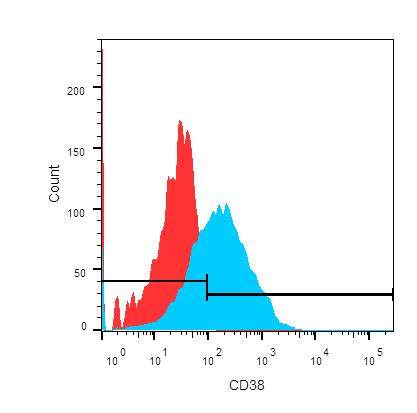
**

CD38low CD38high

KG-1

TF-1

CD38-:47.6% CD38+:52.4%

CD38-:58.7% CD38+:41.3

CD38-:34.5% CD38+:65.5

CD38-:82.9% CD38+:17.1%

CD38

Isotype control

A

B

**Fig. S4.**

Annexin V

PI

TF-1 CD38high

TF-1 CD38low

KG-1 CD38low

KG-1 CD38high

control HHT (20 ng/ml) HHT (40 ng/ml) As_2_O_3_ (1 μM，2 μM for TF-1) HHT (20 ng/ml)+As_2_O_3_ (1 μM) HHT (20 ng/ml)+As_2_O_3_ (2 μM)
