# Supplementary material for: Synergistic killing effects of homoharringtonine and arsenic trioxide on acute myeloid leukemia stem cells and the underlying mechanisms
Source: J Exp Clin Cancer Res. 2019 Jul 15;38:308. doi: 10.1186/s13046-019-1295-8 (PMC6631946; doi:10.1186/s13046-019-1295-8)
Supplement: Supplementary file 7 — Figure S7. RNA sequencing and functional enrichment analysis. KG-1 cells were treated with Homoharringtonine (HHT) and arsenic trioxide (ATO) alone or combined for 6 h and then RNA was isolated, after mRNA sequencing performed, cluster analysis and pathway enrichment analysis were applied. (DOCX 224 kb) [file 13046_2019_1295_MOESM7_ESM.docx]

**
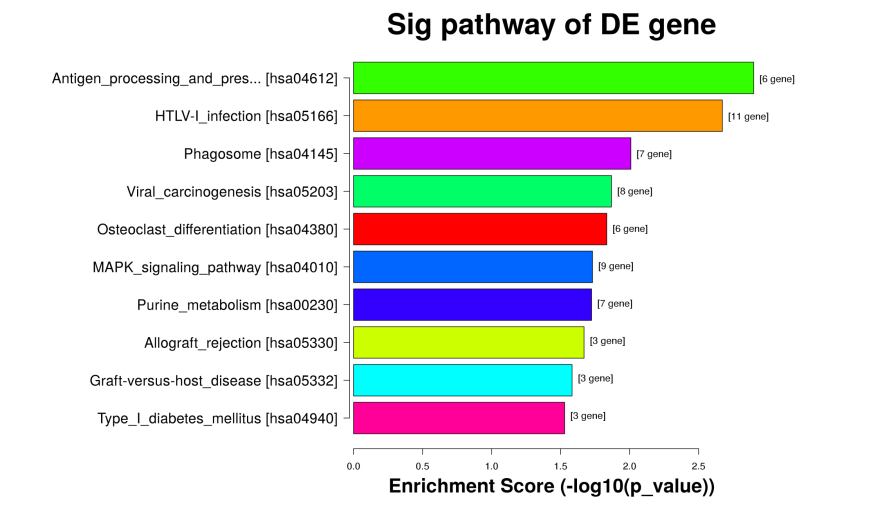

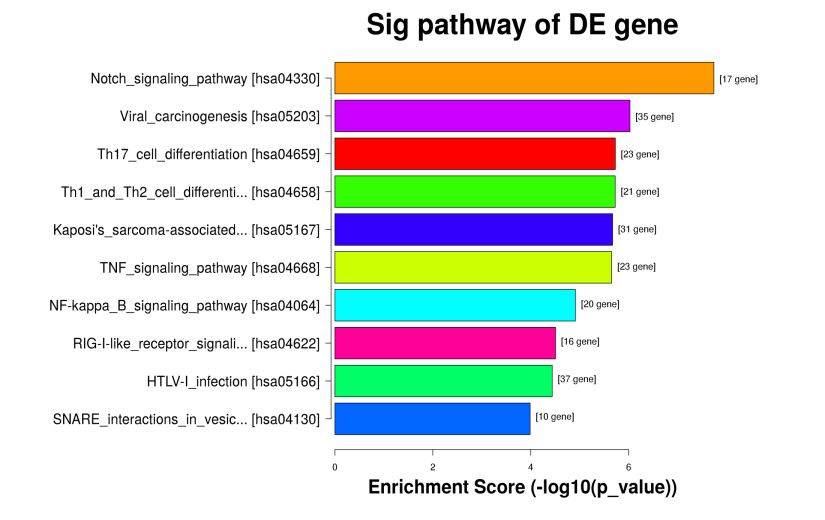
**

A

B

HHT+As2O3 vs HHT downregulation genes

HHT vs control upregulation genes

**
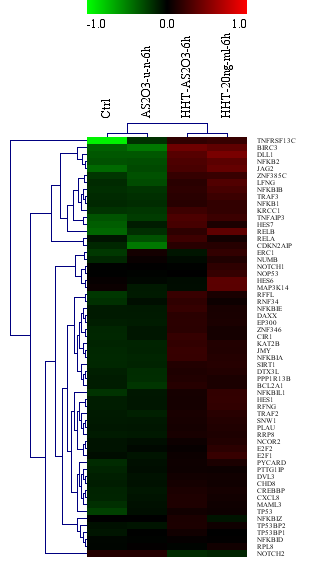

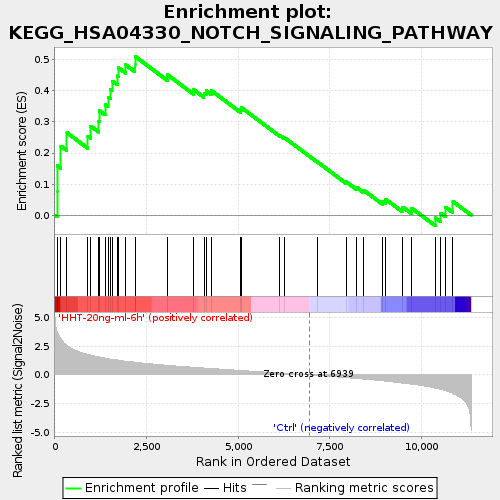

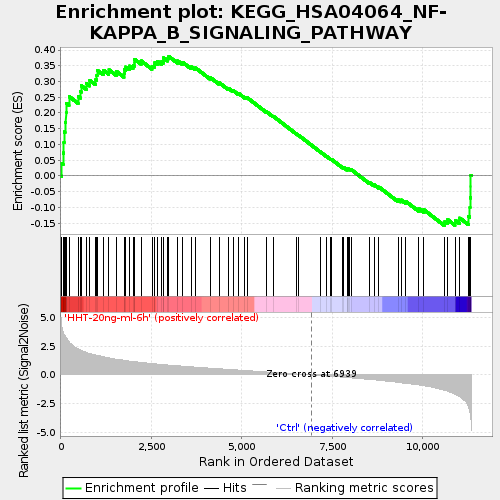
**

p value: 1.22E-05

Enrichent Score: 4.91

p value: 1.82E-08

Enrichent Score: 7.74

**Fig. S7.**
