# Supplementary material for: Synergistic killing effects of homoharringtonine and arsenic trioxide on acute myeloid leukemia stem cells and the underlying mechanisms
Source: J Exp Clin Cancer Res. 2019 Jul 15;38:308. doi: 10.1186/s13046-019-1295-8 (PMC6631946; doi:10.1186/s13046-019-1295-8)
Supplement: Supplementary file 8 — Figure S8. Homoharringtonine (HHT) combined with arsenic trioxide (ATO) alter the expression of P53 and NF-κB2. P53 and NF-κB2 levels were detected in the different groups by confocal laser-scanning microscopy in representative 4% paraformaldehyde-fixed spleens and bone marrow samples from NRG mice. Scale bars: 50 μm (DOCX 1214 kb) [file 13046_2019_1295_MOESM8_ESM.docx]

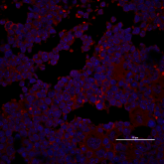

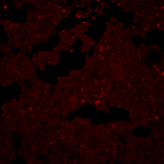

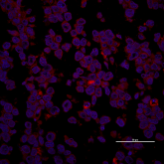

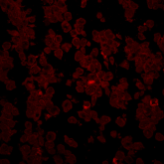

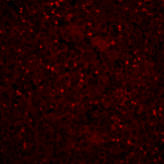

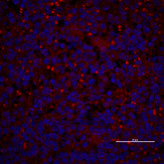

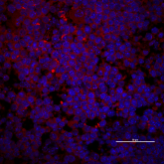

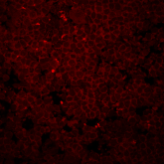

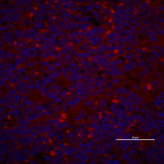

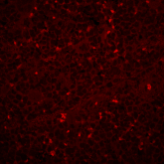

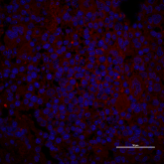

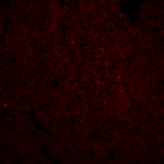


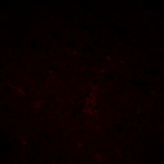

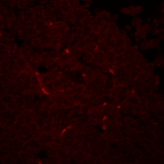

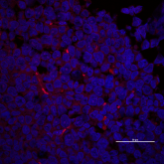

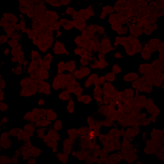

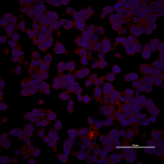


P53

Merge

NF-κB

Merge

Spleens Bone marrows


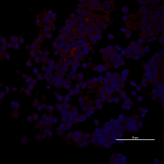

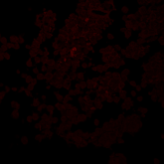

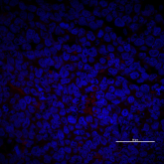

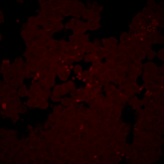

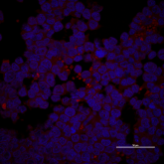

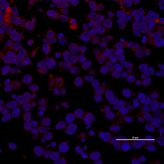

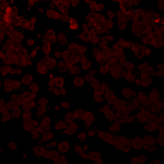

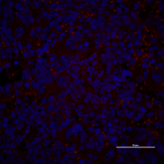

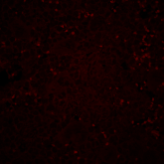

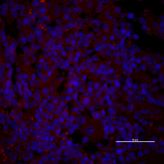

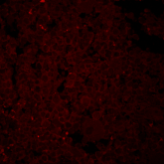

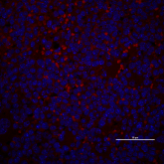

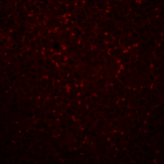

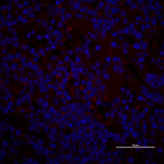

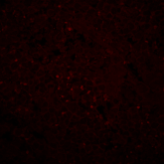


**Fig.S8**

Vehicle HHT As_2_O_3_ HHT+ As_2_O_3_  Vehicle HHT As_2_O_3_ HHT+ As_2_O_3_

(1×PBS) (1×PBS)
