# Supplementary material for: Synergistic killing effects of homoharringtonine and arsenic trioxide on acute myeloid leukemia stem cells and the underlying mechanisms
Source: J Exp Clin Cancer Res. 2019 Jul 15;38:308. doi: 10.1186/s13046-019-1295-8 (PMC6631946; doi:10.1186/s13046-019-1295-8)
Supplement: Supplementary file 9 — Table S1. Patients characteristic (DOCX 27 kb) [file 13046_2019_1295_MOESM9_ESM.docx]

**Table S1. Patients characteristic**

NO. Gender age WBC Hb (g/L) PLT FAB type BM Blasts immune markers karyotype

(*10^9^/L) (*10^9^/L) (%)

1 female 23 1.85 124 168 M0 63.2 CD7, CD117, HLA-DR 46 XX

2 male 29 12.72 138 7 M5 60.3 CD7,HLA-DR, CD33 46 XY

3 male 32 1.14 46 4 M_2_a; 80 MPO,CD99,CD117, t(8;21)

4 female 36 5.57 50 83 M_5_b 78 CD117, CD33,MPO 46 XX

5 female 43 20.57 45 10 M1 72 CD117, CD33, MPO 46 XX

6 female 25 19.6 34 23 M0 69.3 CD7, CD117, MPO 46 XX

7 male 29 27 23 3 M5 59.6 CD33, HLA-DR CD15 46 XY

NO.1-4 were used to FCM (Flow Cytometry) analysis; NO.5-7 were used to synergistic effect. NO.7 were also used for WB.
