# Supplementary material for: Synergistic killing effects of homoharringtonine and arsenic trioxide on acute myeloid leukemia stem cells and the underlying mechanisms
Source: J Exp Clin Cancer Res. 2019 Jul 15;38:308. doi: 10.1186/s13046-019-1295-8 (PMC6631946; doi:10.1186/s13046-019-1295-8)
Supplement: Supplementary file 10 — Table S2. Primer Sequences for PCR (DOCX 18 kb) [file 13046_2019_1295_MOESM10_ESM.docx]

**Table S2. Primer Sequences for PCR**

Gene Primer Sequences

β-actin Forward 5’-GCCAACCGCGAGAAGATGA-3’

Reverse 5’-CATCAGGATGCCAGTGGT-3’

CD34 Forward 5’- ACTCGGTGCGTCTCTCTAGG -3’

Reverse 5’- CCGTGAGACTCTGCTCTGC-3’

CD38 Forward 5’- TTG GGA ACTCAG ACC GTA CCT TG-3’

Reverse 5’- CCA CAC CAT GTGAGG TCA TC-3’

CD96 Forward 5’- ACCACAGTCAAGGTTTTTG-3’

Reverse 5’- CCAGGCTGGAGAAGGTTGG-3’
